# The power of the age standardized incidence rate to discover the gene link between cancer diseases: development of a new epidemiological method to save money, time, and effort for genetic scientists

**DOI:** 10.2147/OTT.S75785

**Published:** 2015-03-31

**Authors:** Ibrahim G Alghamdi, Issam I Hussain, Mohamed S Alghamdi, Mohammed A El-Sheemy

**Affiliations:** 1 School of Life Sciences, University of Lincoln, Lincoln, UK; 2 College of Medicine, University of Al-Baha, Al-Baha, Saudi Arabia; 3 Ministry of Health, General Directorate of Health Affairs Al-Baha, Al-Baha, Saudi Arabia; 4 Lincoln Hospital, Research and Development United, Lincolnshire Hospitals NHS Trust, Lincoln, UK

**Keywords:** epidemiology, medical statistics, direct standardization, England, Saudi Arabia

## Abstract

**Background:**

This study provides an incipient epidemiological rule using the concept of direct method of standardization to determine the genetic link between cancer diseases.

**Methods:**

The overall 8 or 10 years age standardized incidence rate (ASIR) for both cancer diseases, for example (A) and (B) should be calculated for all regions of the country. A line chart should be used to display the overall ASIR trend of both diseases (A and B). Pearson’s correlation can be used to determine the strength of the association between the overall ASIRs of both diseases. The overlap or opposite direction of the overall ASIR trend of both diseases (A and B) should be determined and studied for possible associations between cancer diseases.

**Results:**

If the trend of the overall 8 or 10 years ASIR of a disease (A) follows that of disease (B) in all regions of the country, then the genes of patients with both diseases (A and B) will be highly homogeneous, and they should be studied in the region with the highest and lowest overall ASIR for both diseases (A and B). In addition, if there is an opposite direction or overlapping trend for both diseases (A and B) in certain regions of the country or among specific groups of people with the same demographic characteristics, then the genes of patients will be investigated for both diseases to identify the potential gene link between cancer diseases.

**Conclusion:**

This study revealed that the overall ASIR trends of female breast cancer, prostate cancer, and ovarian cancer are very similar in all regions of Saudi Arabia and England. Our epidemiological evidence helps to save money, time, and effort for testing the potential gene link between cancer diseases.

## Introduction

This study provides a new epidemiological rule to uncover the gene link between cancer diseases. The concept of direct method of standardization for calculating the age standardized incidence rate (ASIR) of a disease is the foundation of our epidemiological rule for this study. Actually, comparing the morbidity and mortality rates in different regions is an essential step in evaluating the health status of an entire population. The crude rates of morbidity and mortality are not suitable for comparison when there are different age structures in different populations. Therefore, either direct or indirect methods of standardization can be used to control the variables that may otherwise confound the observed relationship. However, “age” and “sex” are the most commonly used variables in standardization.^1^–^5^

The gene link was observed between prostate, ovarian, and breast cancer through BRCA1 and specifically BRCA2. Therefore, if there is a person with prostate cancer and another member of his family with breast or ovarian cancer, this could be related to the BRCA1 or BRCA2 gene. The lifetime risk of these cancer diseases was estimated among individuals who have a mutation in BRCA1 and BRCA2. The risk was 40% to 80% for breast cancer, 11% to 40% for ovarian cancer, and up to 39% for prostate cancer.^6^ However, the main objective of this study was to discover a new epidemiological rule that gives the best indicator for proving the gene link between cancer diseases. Furthermore, our epidemiological rule helps scientists in genetics to investigate a certain region or the appropriate person’s characteristics for the gene link between cancer diseases.

## Materials and methods

**Figure 1:**
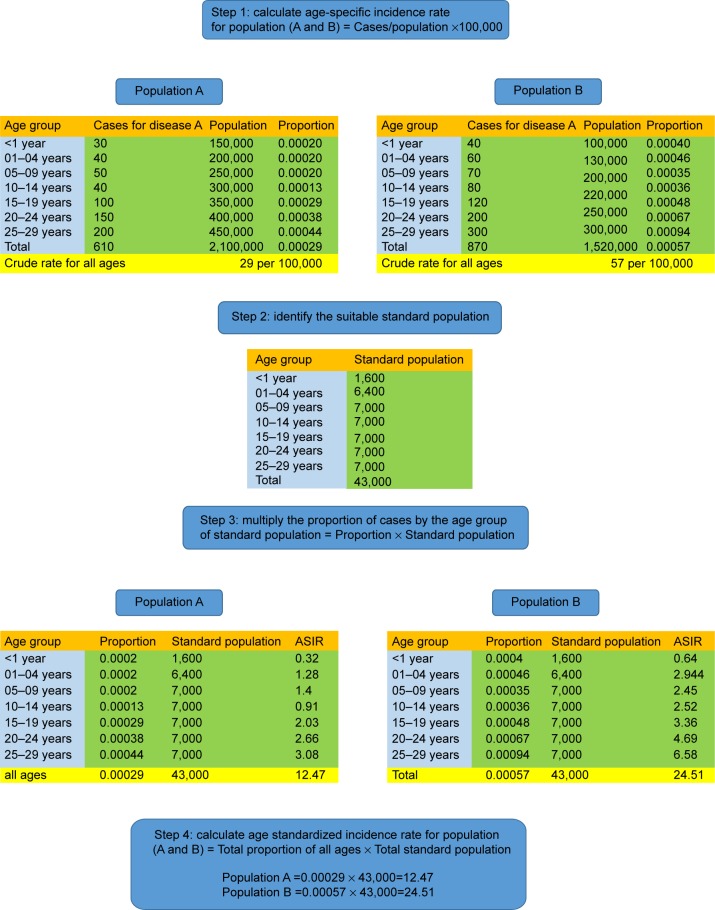
An example of direct age adjustment for calculating age standardized incidence rate. **Abbreviation:** ASIR, age standardized incidence rate.

This section provides an overview of the procedures used in our epidemiological rule to determine the best group of patients for the gene link between cancer diseases. This method can be done after calculating the ASIR of cancer diseases with different persons’ characteristics. The ASIR can be calculated by the direct method of standardization which consists of the following steps (Figure 1):

- Step 1: calculate the age-specific incidence rate for population (A and B).
- Step 2: identify the suitable standard population. This could be European or World standard population.
- Step 3: multiply the proportion of cases by the age group of the standard population.
- Step 4: calculate ASIR for population (A and B) = total proportion of all ages/total standard population.

**Figure 2:**
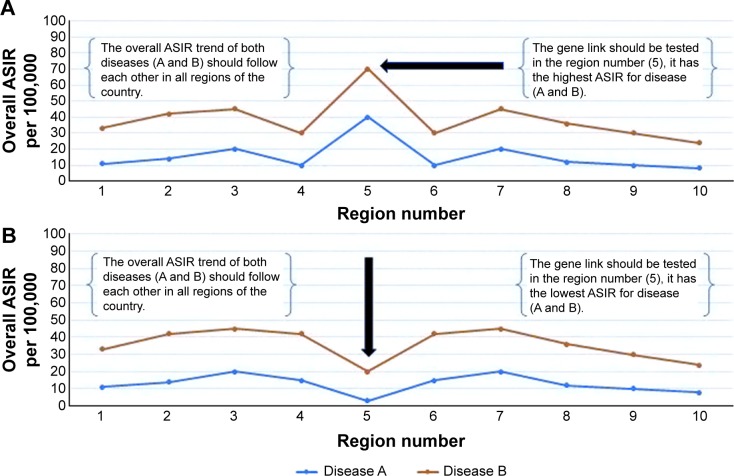
The best geographic area for testing the gene link between cancer diseases. **Notes:** (**A**) The overall ASIR of the diseases, for example (disease A and disease B) during an 8-year period. The region number 5 has the highest rate for both diseases and should be tested for the gene link among patients. The overall ASIR trend of both diseases (A and B) should follow each other in all regions of the country. (**B**) The overall ASIR of the diseases (A and B) during an 8-year period. The region number 5 has the lowest rate for both diseases and should be tested for the gene link among patients. The overall ASIR trend of both diseases (A and B) should be similar in all regions of the country. **Abbreviation:** ASIR, age standardized incidence rate.

**Figure 3:**
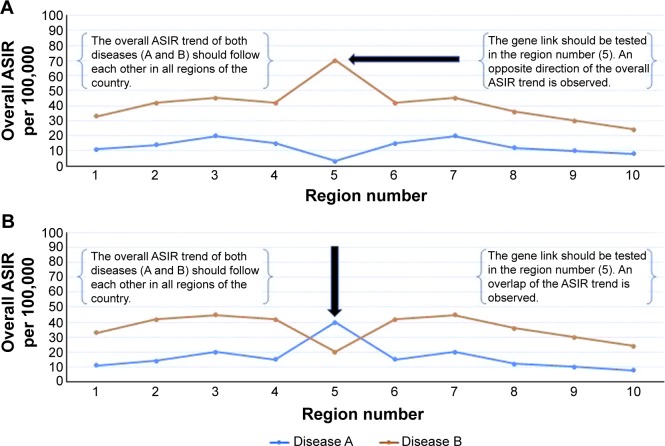
The best geographic area for testing the gene link between cancer diseases. **Notes:** (**A**) The overall ASIR of the diseases, for example (disease A and disease B) during an 8-year period. The region number 5 has an opposite direction of the trend for both diseases and should be tested for the gene link among patients. The overall ASIR trend of both diseases (A and B) should follow each other in all regions of the country. (**B**) The overall ASIR of the disease (A and B) during an 8-year period. The region number 5 has overlap of the trend for both diseases and should be tested for the gene link among patients. The overall ASIR trend of both diseases (A and B) should be similar in all regions of the country. **Abbreviation:** ASIR, age standardized incidence rate.

**Figure 4:**
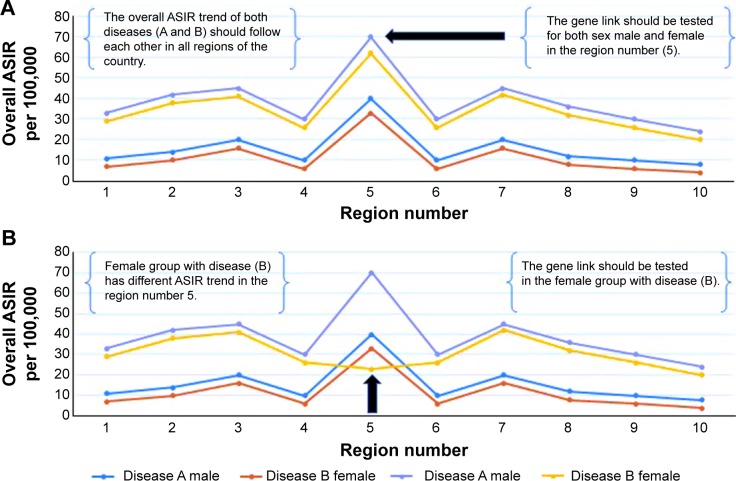
The best geographic area adjusted by sex for testing the gene link between cancer diseases. **Notes:** (**A**) The overall ASIR of the diseases, for example (disease A and disease B), stratified by sex, during an 8-year period. The region number 5 has the highest trend for both diseases among male and female, and should be tested for the gene link in both groups. (**B**) The overall ASIR of the disease (A and B), stratified by sex, during an 8-year period. Female group with disease (B) has different ASIR trend in the region number 5, compared to other groups, the gene link should be investigated for this group. **Abbreviation:** ASIR, age standardized incidence rate.

**Figure 5:**
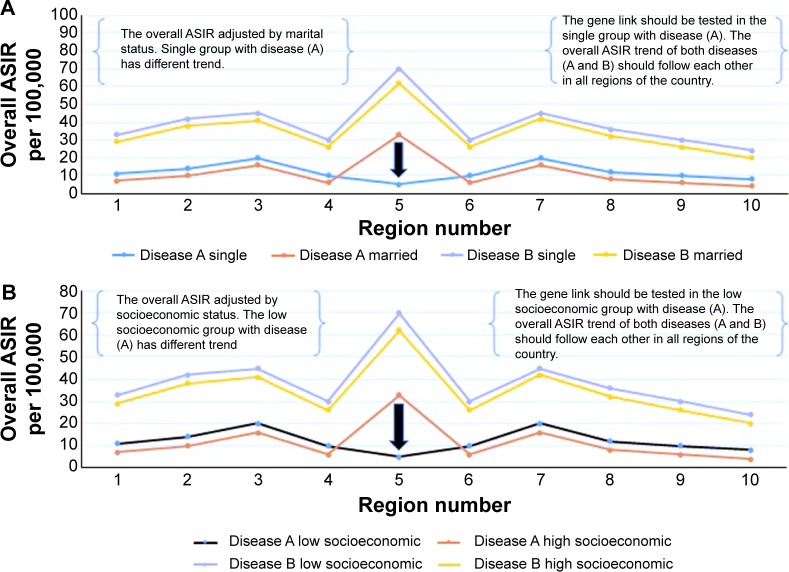
The best geographic area adjusted by socioeconomic and marital status for testing the gene link between cancer diseases. **Notes:** (**A**) The overall ASIR of the diseases, for example (disease A and disease B), stratified by marital status, during an 8-year period. Single group with disease (A) has different ASIR trend in the region number 5, compared to other groups, the gene link should be investigated for this group. (**B**) The overall ASIR of the disease (A and B), stratified by socioeconomic status, during an 8-year period. Low socioeconomic group with disease (A) has different ASIR trend in the region number 5, compared to other groups, the gene link should be investigated for this group. **Abbreviation:** ASIR, age standardized incidence rate.

Our epidemiological rule includes the following stages and should be conducted carefully step-by-step:

- Stage 1:
  ○ A: calculate the overall 8 or 10 years ASIR of disease (A) in all regions of the country.
  ○ B: calculate the overall 8 or 10 years ASIR of disease (B) in all regions of the country.
  ○ C: the calculation can be for multiple diseases with different persons’ characteristics.
  ○ D: the method is efficient for long periods (>8 years) when calculating ASIR.
- Stage 2:
  ○ A: use the line chart to display the overall ASIR of both diseases (A and B).
  ○ B: check the trends of both diseases (A and B) in all regions of the country.
  ○ C: check the correlation of ASIR of both diseases (A and B).
- Stage 3:
  ○ A: if there is a potential relationship between diseases (A and B), the trend of the overall ASIRs for both diseases will be very similar in all regions of the country. This means that there is a gene link between both cancer diseases.
  ○ B: if there is a potential relationship between diseases (A and B), the trend of the overall ASIRs for both diseases will be similar in all regions of the country except one or two areas. The excluded area should be investigated for the gene link between both cancer diseases.
  ○ C: if there is no association between diseases (A and B), the trend of the overall ASIRs for both diseases will not be similar in all regions of the country.
- Stage 4:
  ○ A: investigate the genes of patients with diseases (A and B), when the overall ASIRs for both diseases are very high or very low in a certain region of the country. The correlation between the overall ASIR of the diseases (A and B) is positively strong (Figure 2A and B).
  ○ B: investigate the genes of patients with disease (A and B), when there is an opposite direction or overlap of the trends for both diseases in a certain region of the country. The correlation between the overall ASIR of the diseases (A and B) is negatively strong (Figure 3A and B).
- Stage 5:
  ○ A: sex adjustment can be applied for the overall ASIR of both diseases (A and B). This adjustment helps to concentrate the test on a specific sex group (Figure 4A and B).
  ○ B: socioeconomic adjustment can be applied for the overall ASIR of both diseases (A and B). This adjustment helps to concentrate the gene link test on specific socioeconomic groups (Figure 5A and B).
  ○ C: the adjustment can be done for other persons’ characteristics to save time, money, and efforts when testing the genes of patients.
- Stage 6:
  ○ A: our epidemiological rule can also be used for a single cancer disease with different persons’ characteristics, such as ASIR of stomach cancer among males and females.
  ○ B: the highest and lowest ASIR of cancer disease should be determined in all regions of the particular country.
  ○ C: a case control study should be conducted in the area with the highest and lowest ASIR of cancer disease.
  ○ D: the odds ratio of cancer risk factors should be calculated in both areas with the highest and lowest ASIR.
  ○ E: make a comparison between the odds ratio of cancer risk factors in both areas to justify the real and the strongest risk factor of cancer disease.

## Testing the epidemiological rule on real data from Saudi Arabia

We calculated the overall ASIR of female breast cancer and prostate cancer in Saudi Arabia from 2001 to 2008. The ASIR was stratified by the region and year of diagnosis (Tables 1 and 2). Our epidemiological rule was used to prove the epidemiological association between female breast cancer and prostate cancer in Saudi Arabia. However, the previously mentioned stages were applied step-by-step to check the overall ASIR trend for both female breast cancer and prostate cancer in all regions of Saudi Arabia.

## Testing the epidemiological rule on real data from the United Kingdom

We have collected cancer data from the website of United Kingdom’s Office for National Statistics, and calculated the overall ASIR of female breast cancer, prostate cancer, and ovarian cancer from 2003 to 2012 (Table 3). We have tried to test our epidemiological rule and prove the association between these cancers in all geographic areas of England. Furthermore, we independently tested our methods on the most common cancers in England, which include esophagus cancer, stomach cancer, and pancreatic cancer. The ASIRs were stratified by sex to determine the most suited areas to discover the real risk factor of cancer disease among males and females (Table 4).

## Results of the epidemiological rule in Saudi Arabia

**Figure 6:**
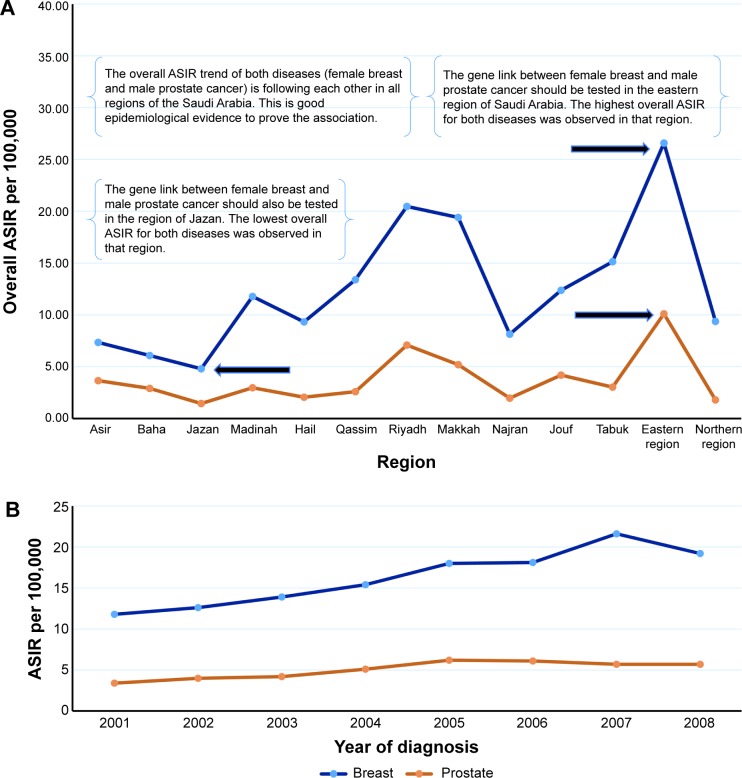
The ASIR of female breast cancer and prostate cancer adjusted by region and year of diagnosis in Saudi Arabia. **Notes:** (**A**) The overall ASIR of female breast and prostate cancer, stratified by region in Saudi Arabia from 2001 to 2008. This method was tested on real data and proved its efficacy to find the best region for the gene link. The eastern region and Jazan were the best places to identify the association between female breast and prostate cancer. (**B**) The overall ASIR of female breast and prostate cancer in Saudi Arabia from 2001 to 2008. The trend of prostate and female breast cancer has the same direction from 2001 to 2008. **Abbreviation:** ASIR, age standardized incidence rate.

In this study, we have reported that the overall ASIR trends of female breast cancer and prostate cancer were very similar and in all regions of Saudi Arabia (Figure 6A). Furthermore, our results confirm that the ASIR of female breast cancer and prostate cancer stratified by the year of diagnosis per 100,000 women and men, indicate a steady increase between 2001 and 2008 (Figure 6B). The highest overall ASIRs of female breast cancer and prostate cancer were observed in the eastern region of Saudi Arabia, while the lowest overall rate was recorded in Jazan. However, the linear trend for ASIR of female breast cancer and prostate cancer was statistically significant from 2001 to 2008, *F* (1, 6) =52,903, *P*<0.001 and *F* (1, 6) =18.047, *P*<0.001. Therefore, the equation for a straight line to predict the annual ASIR of female breast cancer and prostate cancer in Saudi Arabia is:

10.3+(1.33×years)and3.4+(0.4×years). (1)

## Results of the epidemiological rule in England

**Figure 7:**
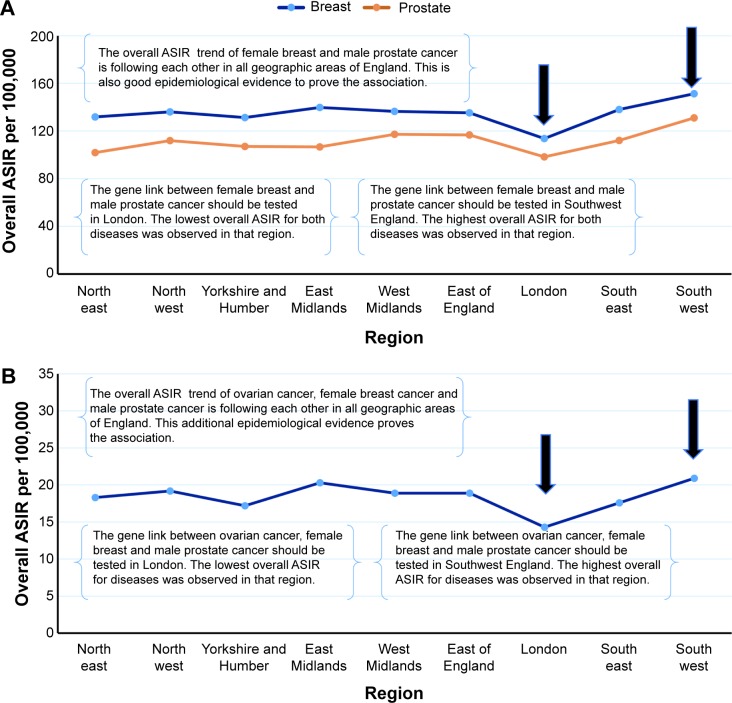
The overall ASIR of female breast cancer, ovarian cancer and prostate cancer adjusted by geographic areas in England, UK. **Notes:** (**A** and **B**) The overall ASIR of female breast and prostate cancer, stratified by region in England from 2003 to 2012. The data were collected from United Kingdom’s Office for National Statistics. **Abbreviation:** ASIR, age standardized incidence rate.

The overall ASIR trends of female breast cancer, prostate cancer, and ovarian cancer during the years 2003 to 2012, were very similar in all regions of England (Figure 7A and B). The highest overall ASIRs of female breast cancer, prostate cancer, and ovarian cancer were observed in the southwest region of England, while the lowest was in London. The most common cancers in the United Kingdom were investigated by our epidemiological method to identify the best geographic areas for studying the real risk factor of cancer disease. The results are mentioned in the next sections.

### Esophagus cancer

**Figure 8:**
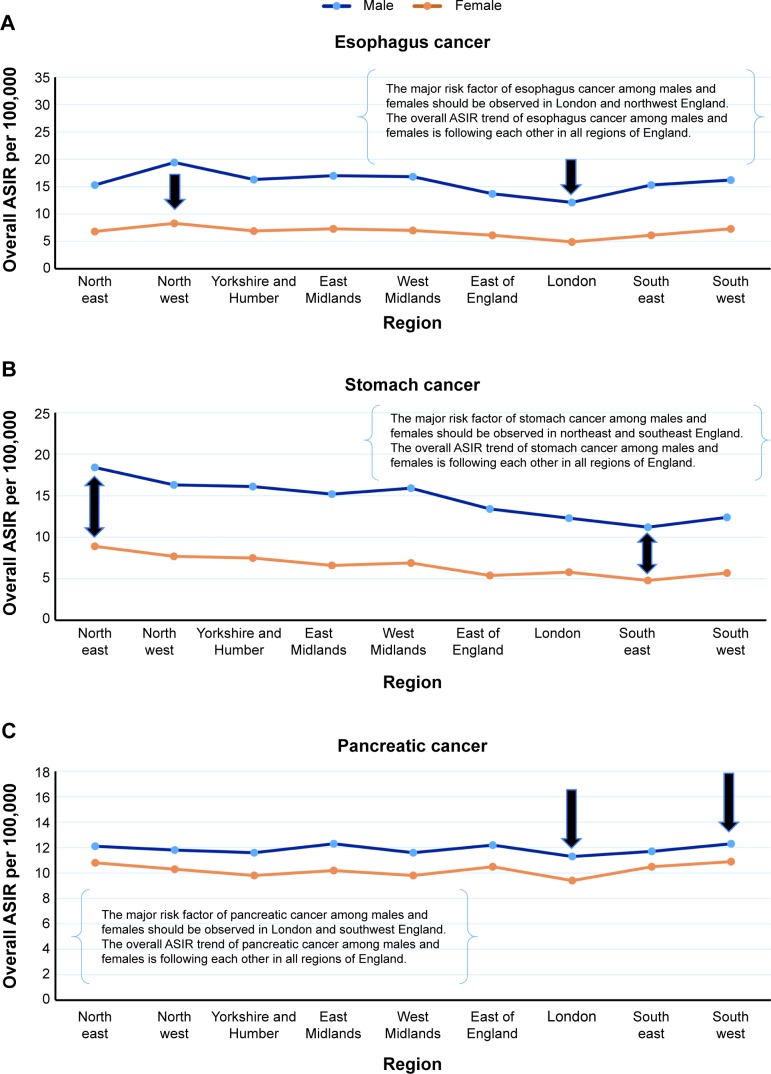
The overall ASIR of esophagus cancer, stomach cancer, and pancreatic cancer adjusted by geographic areas in England, UK. **Notes:** (**A**) The overall ASIR of esophagus cancer among males and females stratified by region in England from 2003 to 2012. (**B**) The overall ASIR of stomach cancer stratified by region in England from 2003 to 2012. (**C**) The overall ASIR of pancreatic cancer stratified by region in England from 2003 to 2012. The data were collected from United Kingdom’s Office for National Statistics. **Abbreviation:** ASIR, age standardized incidence rate.

The overall ASIR trend of esophagus cancer among males and females, was very similar in all geographic areas of England (Figure 8A). The highest overall ASIR of esophagus cancer among males and females was recorded in the northwest region of England, while the lowest was observed in London. Furthermore, there was a very strong correlation between male and female esophagus cancer, *R*=0.96, *P*<0.001.

### Stomach cancer

The overall ASIR trend of stomach cancer among males and females was also very similar in all geographic areas of England (Figure 8B). The highest overall ASIR of stomach cancer among males and females was recorded in the northeast region of England, while the lowest was observed in the southeast. Furthermore, there was a very strong correlation between male and female stomach cancer, *R*=0.88, *P*<0.001.

### Pancreatic cancer

The overall ASIR trend of pancreatic cancer among males and females, was very similar in all geographic areas of England (Figure 8C). The highest overall ASIR of pancreatic cancer among males and females was recorded in the southwest region of England, while the lowest being observed in London. Furthermore, there was a strong correlation between male and female pancreatic cancer, *R*=0.80, *P*<0.001.

## Discussion

The technique of standardization (either direct or indirect) is very important in epidemiology when making comparisons between different demographic structures of populations. The measures of morbidity and mortality could be misleading techniques when comparing populations that differ in their age structure. Therefore, standardization helps in adjusting confounding variables that distort the results of studies. However, we have created a new epidemiological method that helps genetic scientists to identify the best geographic areas to be tested for the gene links of cancer diseases. In addition, our epidemiological rule was based on the direct method of standardization that we used to calculate the ASIR of cancer diseases in different regions of Saudi Arabia and the United Kingdom. So, the effectiveness of this rule in discovering the potential gene link between cancer diseases was proved by using our method.

In this study, we have reported that the overall ASIR trend of female breast cancer and prostate cancer was very similar in all regions of Saudi Arabia. The overall trend of these diseases did not occur by chance in Saudi Arabia during the years 2001–2008. Therefore, the actual trends of the overall ASIRs for female breast cancer and prostate cancer should be similar in all regions and states of the different countries. However, we strongly recommend – based on our method – to investigate the gene link between female breast cancer and prostate cancer in Jazan and the eastern region of Saudi Arabia. In the United Kingdom, the results indicated that the overall ASIR trends of female breast cancer, prostate cancer, and ovarian cancer were very similar in all geographic areas of England. Therefore, we suggest that the gene link between these cancer diseases in London and southwest region of England be tested. However, for testing the gene link of patients in certain regions with multiple diseases, it is important to use our epidemiological rule with different persons’ characteristics such as sex, marital status, socioeconomic status, and race.

Our epidemiological rule proved that the overall ASIR trend of esophagus cancer among males and females, from 2003 to 2012, was very similar in all geographic areas of England. Therefore, we strongly recommend the investigation of the real risk factor of esophagus cancer in London and the northwest region of England. This can be done by conducting a case control study adjusted by sex in both geographical areas. The odds ratio should be compared in these regions to identify the real risk factor for esophagus cancer. Similarly, the overall ASIR trend of stomach cancer among males and females was also very similar in all geographic areas of England, with the best area to identify the real risk factor of this disease among males and females being northeast and southeast England. In addition, the risk factor of pancreatic cancer should be observed in London and southwest England.

Finally, it is challenging to study the gene link and real risk factor of cancer diseases, but through the first step of epidemiology, we can interpret the distribution of cancer diseases in a population according to time, place, and person. This will help to generate a new hypothesis for future analytical observational studies that include case control and cohort studies. Both studies can be conducted to identify the association between the risk factors and cancer diseases. Furthermore, these analytical studies will help in the prevention of cancer diseases by discovering the strongest risk factors that attribute to the increase of ASIR in a specific region of the country.

## Conclusion

This study revealed that the overall ASIR trends of female breast cancer, prostate cancer, and ovarian cancer are very similar in all regions of Saudi Arabia and United Kingdom. The best areas to be tested for the gene link of these diseases in Saudi Arabia is the eastern region and Jazan while for the United Kingdom, it is London and southwest England. The best area in England to be tested for the major risk factor of esophagus cancer among males and females is London and northwest England, while for stomach cancer, the best regions are the northeast and southeast England. In addition, southwest England and London are the best areas for testing the risk factor of pancreatic cancer. Our epidemiological rule may help scientists of genetics to identify the best region for linking the gene of cancer diseases.

## Figures and Tables

**Table 1:** The ASIR of female breast cancer and prostate cancer cases in Saudi Arabia from 2001 to 2008

Year	ASIR per 100,000	
	Female breast cancer	Prostate cancer
	ASIR per 100,000 women	ASIR per 100,000 men
2001	11.8	3.4
2002	12.6	4.0
2003	13.9	4.2
2004	15.4	5.1
2005	18.0	6.2
2006	18.1	6.1
2007	21.6	5.7
2008	19.2	5.7

**Abbreviation:** ASIR, age standardized incidence rate.

**Table 2:** The overall ASIR of female breast cancer and prostate cancer cases in all regions of Saudi Arabia from 2001 to 2008

Geographic area	Overall ASIR per 100,000	
	Female breast cancer	Prostate cancer
	
Saudi Arabia	ASIR per 100,000 women	ASIR per 100,000 men
Asir	7.3	3.7
Baha	6.1	2.9
Jazan	4.8	1.4
Madinah	11.8	3.0
Hail	9.3	2.1
Qassim	13.4	2.6
Riyadh	20.5	7.1
Makkah	19.4	5.2
Najran	8.1	2.0
Jouf	12.4	4.2
Tabuk	15.1	3.0
Eastern region	26.6	10.1
Northern region	9.4	3.9

**Abbreviation:** ASIR, age standardized incidence rate.

**Table 3:** The overall ASIR of female breast cancer, ovarian cancer, and prostate cancer cases in all regions of England from 2003 to 2012

Geographic area	Overall ASIR per 100,000 population		
England	Female breast cancer	Ovarian cancer	Prostate cancer
Northeast	132.3	18.3	100.2
Northwest	136.3	19.2	115.0
Yorkshire and Humber	131.5	17.2	108.8
East Midlands	140.5	20.3	110.5
West Midlands	136.0	18.9	118.8
East of England	134.2	18.9	117.7
London	115.5	14.3	103.8
Southeast	136.7	17.6	112.4
Southwest	147.9	20.9	130.2

**Abbreviation:** ASIR, age standardized incidence rate.

**Table 4:** The overall ASIR of esophagus cancer, stomach cancer, and pancreatic cancer among males and females in all regions of England from 2003 to 2012

Geographic area	Overall ASIR per 100,000 population					
	Esophagus cancer		Stomach cancer		Pancreatic cancer	
England	Male	Female	Male	Female	Male	Female
Northeast	15.3	6.8	18.4	8.9	12.1	10.8
Northwest	19.4	8.3	16.3	7.7	11.8	10.3
Yorkshire and Humber	16.3	6.9	16.1	7.5	11.6	9.8
East Midlands	17	7.3	15.2	6.6	12.3	10.2
West Midlands	16.8	7	15.9	6.9	11.6	9.8
East of England	13.7	6.1	13.4	5.4	12.2	10.5
London	12.1	4.9	12.3	5.8	11.3	9.4
Southeast	15.3	6.1	11.2	4.8	11.7	10.5
Southwest	16.2	7.3	12.4	5.7	12.3	10.9

**Abbreviation:** ASIR, age standardized incidence rate.
